# Editorial: Reviews in veterinary epidemiology and economics

**DOI:** 10.3389/fvets.2024.1503288

**Published:** 2024-10-15

**Authors:** David C. B. Taras, Marta Canuti, Yogesh Chander, Camila Hamond, Dasiel Obregon

**Affiliations:** ^1^Boehringer Ingelheim, Ingelheim, Germany; ^2^Department of Veterinary and Animal Sciences, University of Copenhagen, Copenhagen, Denmark; ^3^Varigen Biosciences Corporation, Madison, WI, United States; ^4^National Veterinary Services Laboratories Diagnostic Bacteriology and Pathology Laboratory, Animal and Plant Health Inspection Service United States Department of Agriculture (USDA), Ames, IA, United States; ^5^School of Environmental Science, University of Guelph, Guelph, ON, Canada

**Keywords:** infectious diseases, host susceptibility, CDS tools, freedom from infection, *Salmonella* Dublin, bovine paratuberculosis

Veterinary epidemiology is crucial in understanding infectious diseases that impact the health of animals and humans and thereby, the economy. This Research Topic aimd to publish review papers on key Research Topics in veterinary infectious diseases updating on recent advances in the field and emphasizing important developments in the fields of diagnostics, prevention, treatment, epidemiology, and ecology. The five papers we present here offer a selective glance at the current state and future direction of veterinary infectious disease research by offering insights into some of the current challenges and potential solutions in this field. They highlight the growing importance of technology in predicting, diagnosing, and managing these diseases.

Interspecies transmission of virus is often the origin of emerging diseases in animals and humans, just recently corroborated by SARS-CoV-2 and influenza virus H5N1. Our ability to prepare effective defense strategies and subsequently control such outbreaks depends on our capacity to predict which hosts might be susceptible to which known viruses. In this context Alberts et al. reviewed a highly diverse variety of machine learning algorithms and bioinformatic approaches that have been used between the years 2000 and 2022 to identify potential virus reservoirs on the basis of influenza and coronavirus genome data. The authors link the heterogeneity of approaches to their exploratory nature in a fast-developing research area of predictive modeling. They point out that data availability is a limiting resource and advise to consider sufficient specificity and quality of the data as a prerequisite for a thorough analysis.

The increased implementation of new technologies in veterinary application in recent years is not limited to machine learning approaches but includes advanced mathematical modeling, e.g., in the form of probabilistic models such as Bayesian networks. Yusuf et al. reviewed the use of these and several other mathematical methodologies by digital clinical decision support (CDS) tools that aim at improving diagnostic and treatment decision-making. Based on studies published between 2017 and 2023 the authors recommend a methodological approach for the development of veterinary CDS tools in lower- and middle-income countries (LMICs), which includes the use of Bayesian algorithms and local expert knowledge. They believe that eventually, digital CDS tools can contribute to improved antimicrobial stewardship practices in areas of high need.

Meletis et al. provided an overview of the most commonly used methods for probability estimation such as scenario trees, Bayesian belief networks, simulation methods, Bayesian prevalence estimation methods, and the STOC free model, which can all be used to substantiate freedom from infection based on surveillance data for the example of non-regulated infectious cattle diseases. By describing the variety of influencing factors and limiting assumptions for the choice of a method the authors aim to provide a guide for choosing an appropriate method in different settings. While currently, design and outcomes of heterogeneous control programs result in a lack of comparability, programs robust against differing modalities can be designed which outcomes are comparable, the so-called output-based surveillance. Reviewing the epidemiological and methodological considerations when designing a surveillance program in an output-based framework represents the second objective of the research.

The two remaining articles in this Research Topic remain with cattle by looking at two epidemiologically important pathogens, *Salmonella* Dublin and *Mycobacterium avium subspecies paratuberculosis*.

Velasquez-Munoz et al. discussed the challenges posed by *Salmonella* Dublin, which can severely affect cattle and human health due to its multi-drug resistant characteristics. They stress the difficulties in controlling and eradicating *S*. Dublin from positive herds, as infection may persist in latent carriers and intermittently be shed into the environment, and provide an overview of the effectiveness of strategies that could be implemented in dairy facilities to prevent and control the disease. The review identifies gaps in the knowledge on regional prevalence estimation, on vaccines for calves, and on the economic impact of outbreaks.

Griss et al. provided an overview on the association between *Mycobacterium avium* subspecies *paratuberculosis* infection and the resulting economic burden of bovine paratuberculosis (PTB). They address the need for accurate estimates of the effects on production associated with the disease, a key requirement for evaluating the benefits of potential control programs. The need for more studies on the association between PTB and, particularly, fertility and meat production and on the associations of the different infection status and changes in productivity is highlighted. The authors point out the lack of studies from certain regions and on cow breeds other than Holsteins. Nevertheless, the authors conclude that evidence-based inputs for the development of economic models for bovine paratuberculosis impact estimations are available.

Collectively, these papers underscore the importance of technology in shaping the future of veterinary epidemiology and economics. They highlight the potential of machine learning and digital clinical decision support tools in predicting, diagnosing, and managing infectious diseases. They also underscore the profound socio-economic implications of infectious diseases in animals, and the need for effective control and prevention strategies. However, much work remains to be done. We need to continue investing in research and development and ensure that the benefits of these advancements are accessible to all, regardless of their geographical location or economic status.

